# Successful tocilizumab-based combination therapy for a case of rapidly progressive adult deep morphea with multiple antiphospholipid antibodies: a case report and literature review

**DOI:** 10.3389/fimmu.2026.1764835

**Published:** 2026-02-11

**Authors:** Ruohan Yu, Lina Zhang, Sheng-Guang Li, Jing Zhang, Ji Li, Yadan Zou, Ting Long, Yanfeng Zhang, Guanjun Yue

**Affiliations:** 1 Department of Rheumatology and Immunology, Peking University International Hospital, Beijing, China; 2 Department of Pathology, Peking University International Hospital, Beijing, China

**Keywords:** antiphospholipid antibodies, case report, deep morphea, interleukin-6 (IL-6), localized scleroderma, tocilizumab

## Abstract

**Background:**

Localized Scleroderma (LoS), particularly aggressive subtypes such as Deep Morphea (morphea profunda), is a rare chronic autoimmune fibrosing disorder that can extend into the subcutaneous tissue, fascia, and muscle. These deep forms carry a high risk of functional impairment. Tocilizumab (TCZ), an anti-interleukin-6 (IL-6) receptor antibody, has emerged as a promising therapy for severe, refractory cases. However, its reported use typically follows the failure of standard immunosuppressive agents like methotrexate (MTX).

**Case presentation:**

We report the case of a 19-year-old male with a rapidly progressive deep morphea of the left lower extremity, with only a two-month history from onset. Initial symptoms included skin hardening, hyperpigmentation, and mild restriction of foot motion. Skin biopsy confirmed deep morphea, showing lymphoplasmacytic inflammation and eosinophilic fibrosis extending into the subcutaneous septa and muscle interstitium. Pre-treatment magnetic resonance imaging (MRI) revealed prominent edema (high T2 signal) in the subcutaneous fat and blurred muscle fascial planes, consistent with active deep inflammation. Uniquely, the patient was seropositive for multiple antiphospholipid antibodies (aPLs), including Lupus Anticoagulant (dRVVT ratio 1.34), anti-phosphatidylserine/prothrombin IgM (143.72 U), β2-glycoprotein I IgM (30.9 CU), and anticardiolipin IgM (28.2 CU). Given the rapid progression and deep tissue involvement, an early intensified combination regimen of TCZ (640 mg IV every 4 weeks), MTX (12.5 mg weekly), high-dose corticosteroids (IV pulses followed by 30 mg/day oral prednisone taper), and prophylactic aspirin (100 mg daily) was initiated. Follow-up MRI at six months showed a marked reduction in the deep tissue edema, correlating with significant clinical improvement in skin induration and tightening by nine months post-treatment. No serious adverse events were observed during follow-up.

**Conclusion:**

This case demonstrates the successful outcome of early TCZ-based combination therapy in rapidly controlling the aggressive inflammatory process of an adult deep morphea. The objective radiological response validates this early intervention strategy, which deviates from the typical second-line use of TCZ. Furthermore, the case highlights a rare but clinically important overlap between severe localized scleroderma and multiple aPL seropositivity.

## 1 Introduction

Localized scleroderma (LoS), also known as morphea, is a rare, chronic autoimmune fibrosing disorder characterized by inflammation and excessive collagen deposition in the skin and underlying soft tissues (1). LoS is classified into various subtypes; Deep Morphea (morphea profunda) and Pansclerotic Morphea (PSM) represent particularly severe, aggressive forms that often involve deep fascia and muscle, potentially leading to joint contractures and functional limitations (2, 3). The linear subtype of LoS (linear morphea), which can extend deep into subcutaneous tissues, is especially associated with significant morbidity (4).

The current gold-standard systemic treatment for active, progressive, or deep-seated LoS is MTX combined with corticosteroids, a regimen whose efficacy has been demonstrated primarily in juvenile localized scleroderma (JLS) clinical trials (5). However, treatment guidelines acknowledge that aggressive or deep forms of LoS may respond inadequately or become refractory to these conventional therapies (6–8).

The pathogenesis of morphea involves complex immune pathways in which T-cell dysregulation and vascular injury lead to fibroblast activation and excessive collagen production. Interleukin-6 (IL-6) is a key pro-fibrotic cytokine implicated in driving fibroblast differentiation and collagen synthesis in sclerotic skin disorders. Consequently, TCZ, a humanized monoclonal antibody against the IL-6 receptor, has been explored in severe, treatment-resistant LoS (9, 10). Existing case series and reports almost exclusively describe TCZ use as a second-line or salvage therapy in patients – typically children – who have failed multiple prior systemic treatments (e.g. MTX and mycophenolate mofetil, MMF) (11, 12).

Here, we present a unique case of rapidly progressive deep morphea in a young adult male, distinguished by the concurrent presence of multiple aPLs. Departing from the standard stepwise treatment approach, this patient was treated with an early, intensified combination regimen including TCZ, MTX, and corticosteroids. This strategy resulted in a rapid and sustained clinical improvement with objective radiologic confirmation. The case supports a proactive use of targeted biologic therapy in severe LoS and highlights the management implications of this unusual serological overlap. In this report, “rapidly progressive” refers to an extensive extension of sclerosis from the calf to the dorsum of the foot and toes within only two months from onset, with early functional limitation.

## 2 Case presentation

### 2.1 Clinical presentation and initial assessment

A 19-year-old man presented at Month 0 with a two-month history of rapidly progressive skin tightening and hyperpigmentation on his left lower leg. The initial lesion, which appeared approximately two months prior to presentation on the lateral aspect of the left calf, was characterized by indurated, hyperpigmented skin with localized atrophy. It quickly spread distally to involve the dorsum of the left foot and the third and fourth toes, leading to mild restriction of left foot flexion and extension. The patient’s past medical history was unremarkable aside from allergic rhinitis. He denied any systemic symptoms suggestive of systemic sclerosis (SSc) – such as fever, Raynaud’s phenomenon, arthritis, myalgias, dysphagia, or any signs of internal organ involvement. There was no sclerodactyly or nailfold capillary abnormality.

**Figure 1:**
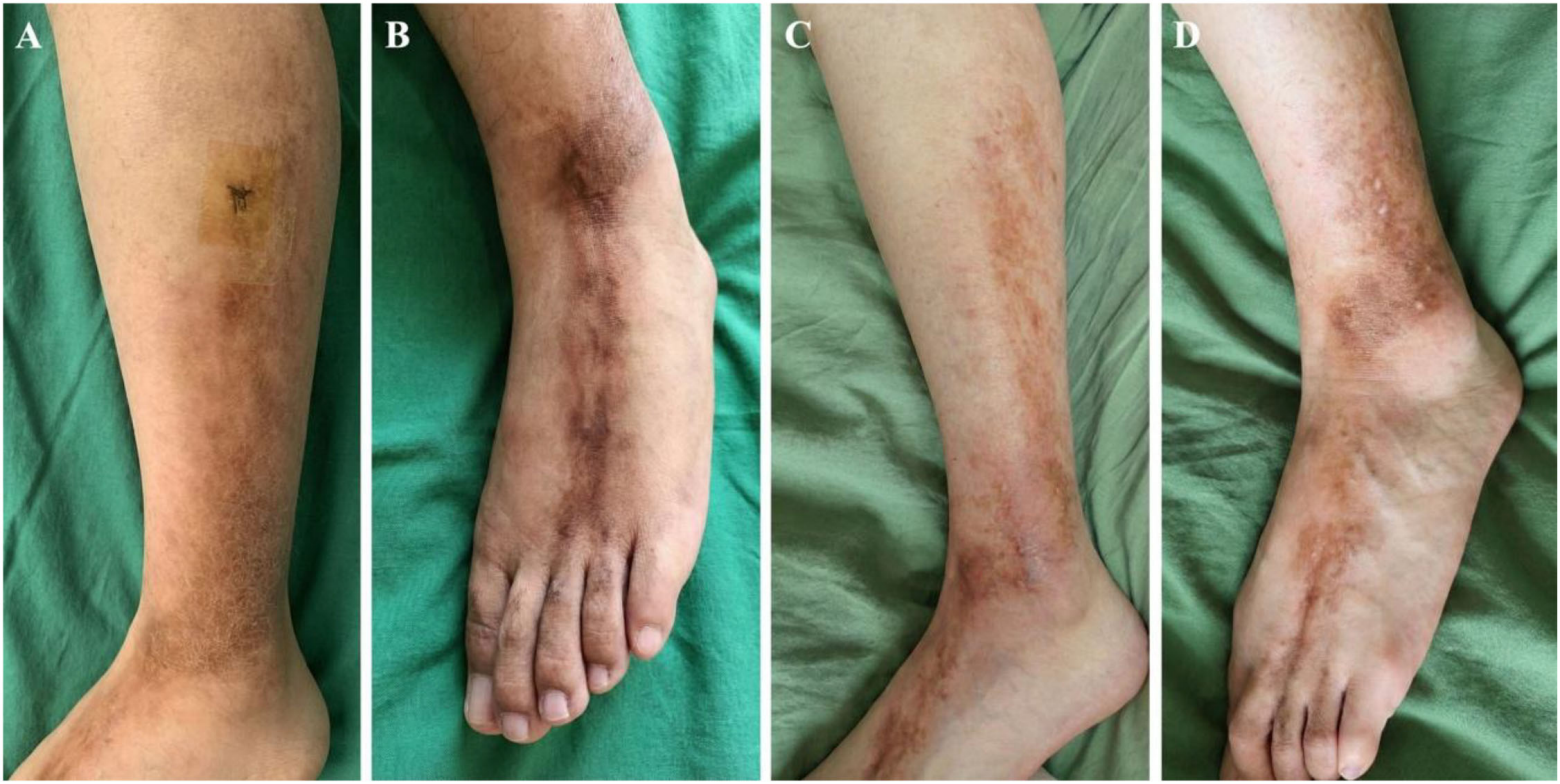
Clinical manifestation and follow-up. **(A)** The skin on the outer side of the left lower leg is firm, swollen, and accompanied by pigmentation (Baseline, Month 0). **(B)** The skin on the sole of the left foot and the skin of the third and fourth toes are hard and tight, accompanied by pigmentation and local skin atrophy (Baseline, Month 0). **(C, D)** The tightness of the skin on the left lower limb, pigmentation, and local skin atrophy have significantly improved (Month 9).

On physical examination, the affected skin over the left lateral lower leg, the dorsum of the left foot, and the third and fourth toes was firm, swollen, hyperpigmented, and showed areas of local depression consistent with atrophy, following a linear band-like distribution (**Figures 1A, B**).

### 2.2 Diagnostic investigations

Routine laboratory tests, including complete blood count, C-reactive protein (CRP), erythrocyte sedimentation rate (ESR), immunoglobulin levels, and hepatic/renal function, were all within normal limits. Crucially, the Antinuclear Antibody (ANA) test was negative, helping to differentiate the condition from SSc. Anti-topoisomerase I (Scl-70) and anticentromere antibodies were also negative.

### 2.3 Unique serological profile

Comprehensive serological evaluation revealed the presence of multiple aPLs. The patient tested positive for lupus anticoagulant (LA; dilute Russell viper venom time ratio 1.34) and had elevated anti-phosphatidylserine/prothrombin IgM (143.72 U), β2-glycoprotein I IgM (30.9 CU), and anticardiolipin IgM (28.2 CU). Although he did not meet the full clinical criteria for antiphospholipid syndrome (APS) (e.g., no thrombotic events or pregnancy morbidity), this profile indicated a state of significant autoimmune-mediated vascular activation. He had no clinical features suggestive of systemic lupus erythematosus and did not fulfill classification criteria for SLE.

### 2.4 Imaging and histopathology confirming deep morphea

#### 2.4.1 High-frequency ultrasound (baseline, month 0)

Ultrasound of the lesion confirmed the depth and severity of involvement. The affected skin was markedly thickened (~3.8 mm thickness, compared to normal skin) with increased echogenicity and indistinct borders between the dermis and subcutaneous tissue. Doppler signal was increased in the lesion, indicating active inflammation.

#### 2.4.2 Magnetic resonance imaging (baseline, month 0)

**Figure 2:**
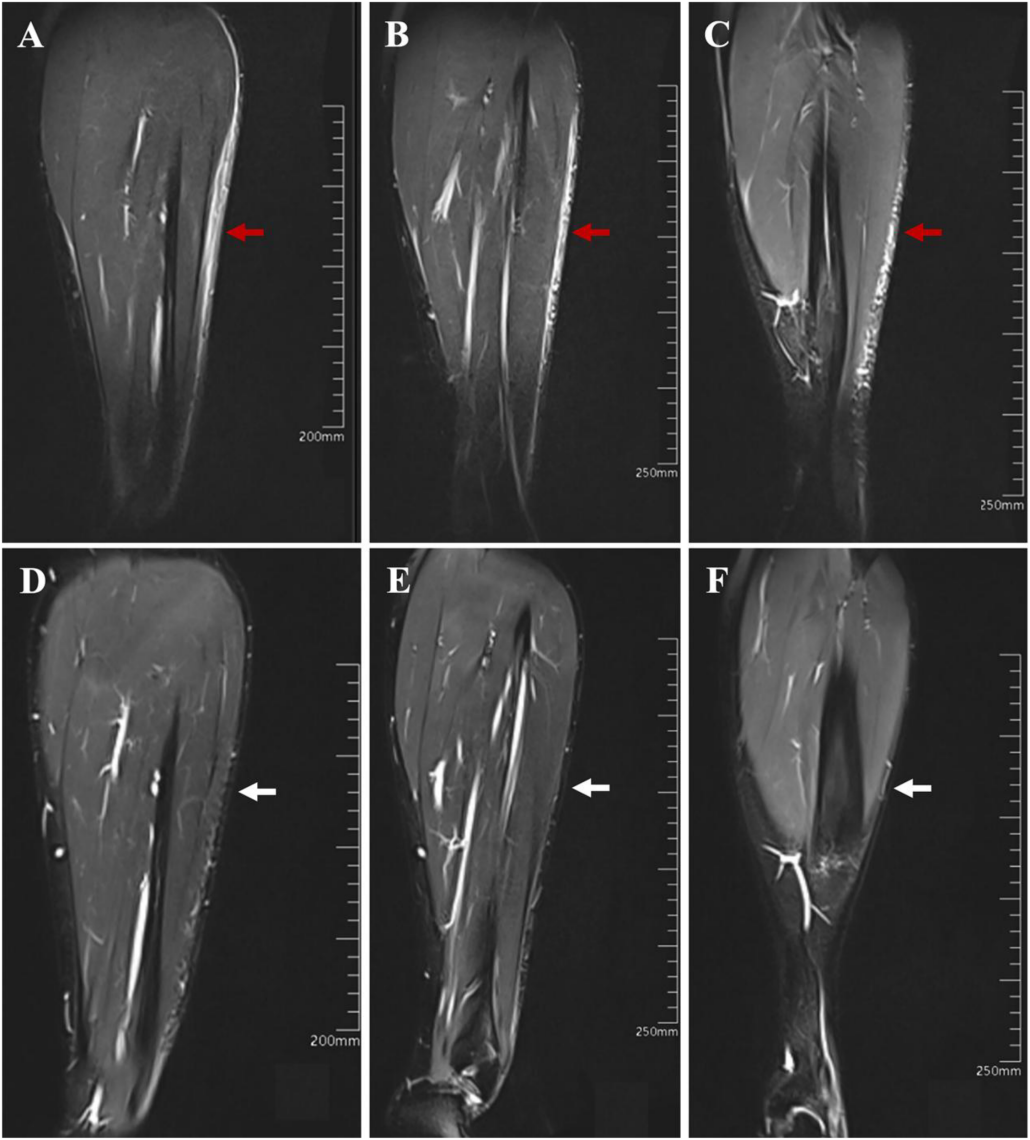
Magnetic resonance imaging findings of the left lower leg. **(A–C)**. Baseline MRI scan (Baseline, Month 0) showing abnormal signals (high signal on fat-suppressed images) in the subcutaneous fat layer and blurred muscle spaces, indicating active Deep Morphea (red arrows). **(D–F)**. Follow-up MRI scan (Month 6) showing significant reduction of high signal shadows in the subcutaneous fat layer (marked reduction, white arrows), consistent with decreased inflammatory activity after 6 months of tocilizumab-based therapy.

Baseline MRI of the left lower leg revealed characteristic findings of deep morphea. T2-weighted fat-suppressed images showed extensive edema in the subcutaneous fat and fuzzy, ill-defined septa within the muscle compartments, reflecting active inflammation extending into deep fascia and muscle (**Figures 2A–C**). These imaging findings provided clear evidence of deep tissue involvement. (MRI is considered a valuable modality for diagnosing and monitoring deep LoS, as recommended by current consensus guidelines). Notably, diffuse circumferential fascial thickening was not observed.

#### 2.4.3 Histopathology (deep skin biopsy of left lower leg)

**Figure 3:**
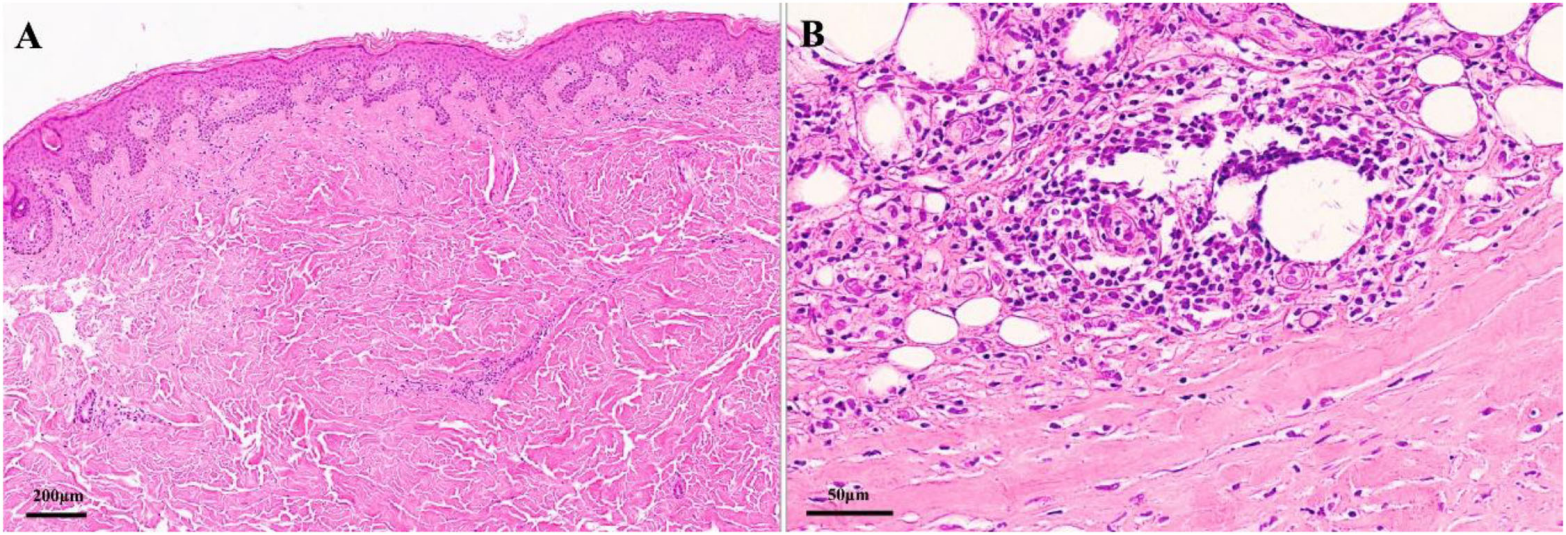
Pathological manifestation of skin biopsy from the left lower leg. **(A)** The dermis shows collagen tissue hyperplasia, partially accompanied by hyaline degeneration. A small number of lymphomonocytes and plasma cells are observed around skin appendages and small blood vessels (4X). **(B)** The subcutaneous adipose tissue exhibits a moderate amount of lymphomonocytes, plasma cells, and histocytes infiltrating between the lobules and within the adipose septa (20X).

Histopathological examination of an excisional biopsy encompassing skin and subcutis corroborated the clinical and imaging findings of deep morphea (linear scleroderma). Key features included fibrous hyperplasia and chronic inflammatory cell infiltration in both the dermis and subcutaneous adipose tissue (**Figure 3**). There was no significant fat necrosis or vasculitis. These findings confirmed active inflammation and sclerosis extending deep into subcutaneous tissue.

### 2.5 Treatment and follow-up

**Figure 4:**
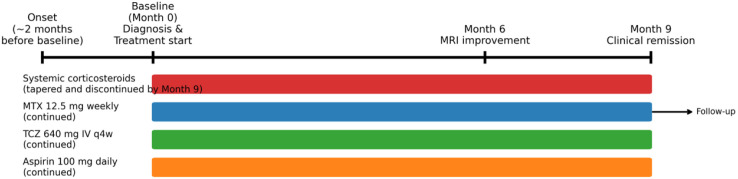
Treatment timeline. A schematic timeline summarizing the main treatment components and key outcomes using relative time points (Month 0, Month 6, Month 9).

Given the aggressive, rapidly progressive nature of the disease and the involvement of deep structures (with high risk for permanent damage), an early intensified multi-target combination therapy was initiated at baseline (Month 0): No concomitant topical therapy was used. A schematic timeline of the treatment course and responses is provided in **Figure 4**.

#### 2.5.1 Corticosteroids

Methylprednisolone pulse therapy (40 mg IV once daily for 3 days) was administered, followed by high-dose oral prednisone (30 mg once daily) with a gradual taper in the ensuing months. Oral prednisone was gradually tapered during follow-up and discontinued by Month 9.

#### 2.5.2 Conventional DMARD

MTX 12.5 mg weekly (subcutaneously) was started as a steroid-sparing immunosuppressant.

#### 2.5.3 Targeted biologic

TCZ 640 mg intravenously every 4 weeks was introduced to directly inhibit IL-6-mediated fibrotic inflammation.

#### 2.5.4 Vascular prophylaxis

Low-dose aspirin (100 mg once daily) was added in light of the patient’s strong aPL positivity, as a precaution against potential thrombotic complications.

#### 2.5.5 Therapeutic response

The patient exhibited a swift and sustained response to therapy:

- Radiological Response (6 months): A follow-up MRI at Month 6 (approximately 6 months after treatment initiation) showed objective evidence of improvement. The previously noted high-signal inflammatory edema in the subcutaneous tissue had markedly diminished, and no abnormal signal remained in the muscle planes (**Figures 2D–F**). This indicates a significant reduction of deep inflammatory activity. Clinically, by Month 6, no further extension of the lesions was observed and skin induration had begun to soften.
- Clinical Response (9 months): By Month 9, the skin tightness, induration, and hyperpigmentation of the left lower leg and foot had significantly improved (**Figures 1C, D**). The affected areas became noticeably softer with improved elasticity, and the patient’s range of motion in the left foot returned to normal. At Month 9, the patient remained on TCZ and MTX without relapse and was off systemic corticosteroids.

This rapid objective and clinical improvement suggested that the aggressive inflammatory process was effectively controlled, likely preventing long-term tissue damage and functional loss.

## 3 Discussion

This case highlights the presentation and successful management of a severe, rapidly progressive deep morphea in an adult patient, distinguished by the presence of multiple aPLs. Remarkably, disease remission was achieved through an early, aggressive combination regimen incorporating TCZ – a biologic therapy that is typically reserved for refractory cases.

### 3.1 Phenotype and diagnostic assessment of deep morphea

Deep morphea (morphea profunda) is characterized by inflammation and sclerosis extending into the deep dermis, subcutaneous fat, and sometimes the underlying fascia or muscle. This subtype of LoS is associated with significant morbidity, particularly when it affects the extremities, as it can cause joint contractures, muscle atrophy, and permanent functional deficits (13). In our patient, the diagnosis of deep morphea was firmly established by histopathology and, critically, by baseline MRI, which demonstrated inflammatory edema tracking along the fat lobules and muscle septa (14). The use of advanced imaging techniques is crucial for deep variants of LoS. MRI, in particular, is highly effective for visualizing the full extent of tissue involvement and detecting active inflammation (edema and contrast enhancement), thereby aiding in both initial assessment and monitoring of treatment response (15). In this case, a 6-month follow-up MRI showing marked reduction of subcutaneous edema provided objective evidence that the therapeutic intervention successfully suppressed the deep inflammatory process. Although eosinophilic fasciitis can show fascial signal abnormalities on MRI, it typically demonstrates more diffuse and symmetric fascial thickening and enhancement (16); this pattern was not observed in our patient, and peripheral eosinophilia was absent.

### 3.2 Rationale for early targeted therapy

Current therapeutic consensus recommends systemic MTX (often combined with corticosteroids) as first-line treatment for aggressive or high-risk LoS, in order to halt disease progression and prevent irreversible damage (6, 7). However, the efficacy of these conventional therapies in severe, deep-seated adult morphea is variable and often suboptimal. In our patient, the combination of rapid progression, deep tissue involvement, and impending functional impairment justified immediate escalation to targeted biologic therapy rather than awaiting failure of MTX alone. This approach aligns with a “treat-to-target” philosophy, aiming to aggressively quench the active inflammation and fibrotic cascade before irreversible fibrosis occurs. Based on this experience, early TCZ may be considered in patients with rapid disease progression, objective evidence of deep inflammatory activity on MRI, and/or imminent risk of functional impairment.

IL-6 plays an integral role in LoS pathogenesis by promoting fibroblast activation and collagen deposition (17–19). TCZ interrupts this pathway by blocking IL-6 signaling. While TCZ has predominantly been reported in severe or refractory cases of LoS (especially in pediatric patients who failed multiple immunosuppressants) (11, 12), our case demonstrates the benefit of integrating TCZ early in the treatment course. Using such a potent agent during the active inflammatory phase likely contributed to the swift remission, supporting the idea that timely intervention with targeted therapy can prevent the progression to crippling fibrosis. Indeed, the successful outcome in this case suggests that TCZ can be a justifiable first-line adjunct in selected aggressive cases of adult deep morphea, representing a potential advance in clinical practice. Notably, both radiological improvement and clear clinical softening were evident by 6 months, whereas most published TCZ cases were treated after prolonged refractory disease (11, 12, 20–23). Nevertheless, intensive combination therapy should be individualized and closely monitored, and it should not be interpreted as universally applicable to all cases of deep morphea.

To date, approximately 26 cases of localized scleroderma treated with TCZ have been reported in the literature, nearly all as salvage therapy after failure of MTX and other immunosuppressives. These reported cases have occurred predominantly in pediatric patients (11, 20–23), most of whom had long-standing, severe disease (often linear, generalized, or pansclerotic subtypes) with extracutaneous or even neurologic/ocular involvement (24, 25), and TCZ was usually added on top of MTX or MMF as rescue therapy, leading to at least partial clinical improvement in the great majority of cases (**Table 1**). In contrast, only a few adult patients with refractory generalized or pansclerotic morphea have been described (9, 10, 24), again receiving TCZ late in the disease course as second- or third-line treatment. Taken together, the cases summarized in **Table 1** indicate that TCZ has mainly been used as a “last-resort” option in deeply fibrosing, treatment-resistant morphea. Notably, our patient is one of the first adults with deep morphea to receive TCZ as an early-line component of combination therapy, and his case is further distinguished by the unique coexistence of multiple aPL positivity. Other biologics (e.g., abatacept and rituximab) and emerging targeted therapies have also been reported for severe/refractory LoS, but evidence remains limited (18, 19).

**Table 1:** Comparative summary of severe localized scleroderma cases treated with tocilizumab.

First author / (Reference)	Publication year	Sex	Age at onset (Years)	Disease duration (Months)	Disease subtype	Major presentation	Disease severity/Special features	Pathology findings	Primary treatment regimen	Outcome / Prognosis
Foeldvari et al. Pt 1/ (20)	2017	F	9	53	Parry Romberg syndrome	Facial atrophy (PRS)	Refractory (MTX + MMF failure), JLS	NR	TCZ monotherapy	mLoSSI decreased, Facial atrophy stable
Foeldvari et al. Pt 2/ (20)	2017	NR	10	12	Linear scleroderma	Extracutaneous activity	Refractory (MTX failure), JLS	NR	TCZ + MTX	mLoSSI decreased, Extracutaneous activity decreased
Foeldvari et al. Pt 3/ (20)	2017	NR	5	17	Generalized	Arthritis	Refractory (MTX+MMF failure), JLS	NR	TCZ + MTX + Prednisone	mLoSSI decreased, Arthritis decreased
Foeldvari et al. Pt 4/ (20)	2017	NR	2	24	Generalized	Arthritis	Refractory (MTX+MMF failure), JLS	NR	TCZ + Tacrolimus + Prednisone	mLoSSI decreased, Arthritis decreased
Foeldvari et al. Pt 5/ (20)	2017	NR	12	168	Limited/Morphea	N/A	Refractory (MTX+MMF, Abatacept failure), JLS, High MRSS (12)	NR	TCZ + MTX	mLoSSI decreased
Foeldvari et al. Pt 6/ (20)	2017	NR	4	21	Linear scleroderma	Arthritis	Refractory (MTX+MMF, Anti-TNF failure), JLS	NR	TCZ + MMF	mLoSSI decreased, Arthritis decreased
Foeldvari et al. Pt 7/ (20)	2017	NR	10	120	Mixed subtype	Arthritis	Refractory (MTX+MMF failure), JLS	NR	TCZ monotherapy	mLoSSI decreased
Foeldvari et al. Pt 8/ (20)	2017	NR	4	2	Linear scleroderma	N/A	Refractory (MTX failure), JLS	NR	TCZ monotherapy	mLoSSI stable, Facial atrophy stable
Foeldvari et al. Pt 9/ (20)	2017	NR	7	6	Linear scleroderma	Arthritis, High MRSS	Refractory (MTX failure), JLS	NR	TCZ + MTX	mLoSSI decreased (from 47 to 40)
Foeldvari et al. Pt 10/ (20)	2017	NR	2	5	Generalized	N/A	Refractory (MTX+MMF failure), JLS	NR	TCZ + MTX	mLoSSI decreased
Foeldvari et al. Pt 11/ (20)	2017	NR	2	108	Morphea en coup de sabre	N/A	Refractory (MTX failure), JLS	NR	TCZ + MTX	mLoSSI stable
Martini et al. Pt 1/ (21)	2017	F	16	NR	Pansclerotic Morphea	Severe full-thickness skin involvement	Refractory (MTX, MMF, Imatinib failure), Severe JLS	NR	TCZ	Partial Remission (PR)
Martini et al. Pt 2/ (21)	2017	M	4	NR	Pansclerotic Morphea	Severe full-thickness skin involvement	Refractory (MTX, MMF failure), Severe JLS	NR	TCZ	Partial Remission (PR)
Lythgoe et al. Pt 1/ (11)	2018	NR	12	124	Linear	Right shin lesion	Severe, long-standing, Refractory (MTX failure)	NR	TCZ + MMF	PGA-A improved from 2.5 to 0.5 (6 mos)
Lythgoe et al. Pt 2/ (11)	2018	NR	9	60	Deep Linear	Left leg length discrepancy, joint contractures, bone changes	Severe, Deep, Congenital, Refractory (MTX failure)	NR	TCZ + MMF (MMF started later)	PGA-A improved from 8.0 to 1.0 (12 mos)
Lythgoe et al. Pt 3/ (11)	2018	NR	6	42	Linear	Right leg and face lesions (HFA)	Associated Uveitis, Refractory	NR	TCZ + MMF + IV MP	PGA-A improved from 8.0 to 2.5 (12 mos)
Lythgoe et al. Pt 4/ (11)	2018	NR	13	22	Linear	Right arm, chest, back lesion	Severe, Refractory (MTX failure)	NR	TCZ monotherapy	PGA-A improved from 8.0 to 0.5 (12 mos)
Lythgoe et al. Pt 5/ (11)	2018	NR	13	59	Generalized	Face, chest, left arm, trunk	Severe, Refractory (MTX failure)	NR	TCZ monotherapy	PGA-A improved from 6.0 to 0.5 (12 mos)
Zhang et al./ (22)	2019	F	6	NR	Pansclerotic Morphea	Extensive, circumferential sclerosis	Extremely Rare, Severe, Refractory (Traditional therapy failure)	NR	Tocilizumab	Rapid response; significant improvement
Magro et al./ (24)	2019	F	29	NR	Linear scleroderma "en coup de sabre" (LSES)	Fronto-parietal induration	Intractable neurological complications, Severe CNS involvement	MxA, C4d, C5b-9, IgG deposition in brain microvasculature (Endotheliopathy)	TCZ	NR (Study focused on pathology/ mechanism)
Osminina et al./ (25)	2020	NR	NR	NR	Scleroderma "en coup de sabre" (LSES)	Craniofacial lesions	Associated Epilepsy and Uveitis (CNS/Extracutaneous involvement)	NR	TCZ	Successfully treated
Ventéjou et al. / (23)	2021	F	8	7	Pansclerotic Morphea (PSM)	Generalized wooden hard, infiltrated skin (mRSS = 29/51), restricted mobility of back, shoulders, and hips	Severe clinical status. Possible streptococcal infection prior to onset. ANA, RF, APLA negative. MRI showed oedema of muscle fasciae	Enlarged dermis, coarse hypertrophic collagen fibers invading hypodermis; sparse perivascular lymphohistiocytic infiltrate; minimal mucin	TCZ (IV 10 mg/kg, then SC 4.5 mg/kg/w) + Prednisolone pulse/oral Prednisone + MTX (16.5 mg/m2/w)	Rapid, complete, and sustained healing. mRSS decreased from 29 to 0/51 after 12 months
Lonowski et al. Pt 1/ (9)	2022	F	41	NR	Generalized morphea	N/A	Refractory	NR	TCZ (162mg sc)	Improved (Median response: 3 months)
Lonowski et al. Pt 2/ (9)	2022	F	69	NR	Generalized morphea	N/A	Refractory	NR	TCZ (162mg sc)	Improved (Median response: 3 months)
Lonowski et al. Pt 3/ (9)	2022	F	50	NR	Linear morphea	N/A	Refractory	NR	TCZ	Improved (Median response: 3 months)
Saraswat et al. / (10)	2024	M	32	9	Pansclerotic Morphea (PSM)	Progressive weakness in both hands; skin tightening on forearms; hyperpigmented, atrophic plaques on shoulder/forearm	Disabling and refractory PSM. Precipitated 2 weeks after COVID-19 infection. Refractory to 6 months of MTX + CS. ANA weakly positive (1:100)	Prominent sclerodermoid changes; thickening and enhancement of skin/subcutaneous/intermuscular fascia on MRI	Corticosteroids + MTX (failed for 6 months); then TCZ (IV 8 mg/kg Q4W)	Excellent outcome. Skin softened, increased range of movement in shoulder/MCP joints after 3 doses of TCZ
Present Case	2026	M	19	2	Deep Morphea / Linear Scleroderma	Left lower leg/foot sclerosis, mild restriction of foot function	Rapidly progressive, Active (Ultrasound: 3.8mm thick, increased flow), Multiple APLA Positive	Lymphocyte, histiocyte, plasma cell infiltration in deep tissue, eosinophilic swelling, suggestive of deep Morphea/Linear Scleroderma	TCZ + MTX + Prednisone + Aspirin	Significant improvement in skin induration and MRI activity

### 3.3 Clinical significance of antiphospholipid antibody overlap

The co-occurrence of LoS with multiple positive aPLs in this patient is an exceptionally rare finding (26, 27). LoS is generally not associated with the systemic microvascular pathology seen in SSc; however, the presence of aPLs denotes a prothrombotic and vasculopathic state. It is conceivable that the aggressive nature of our patient’s morphea was exacerbated – or at least influenced – by an underlying vascular insult mediated by these autoantibodies (28). This consideration influenced our management: we incorporated low-dose aspirin as a preventative measure against thrombotic events (7), given the significant aPL profile. Such an approach (combining immunosuppression with vascular prophylaxis) is uncommon in LoS and highlights the importance of tailoring therapy to individual patient risk factors.

This case underlines the need for thorough serological evaluation in severe or atypical presentations of localized scleroderma. In patients with unusually aggressive disease courses or features suggestive of systemic involvement, screening for concurrent autoimmune markers (e.g., aPLs) may uncover overlaps that have implications for both prognosis and treatment. Identifying an aPL overlap in our patient guided the addition of antithrombotic prophylaxis and alerts clinicians to monitor for potential vascular complications. More broadly, recognizing such overlaps can facilitate a multidisciplinary management approach and inform prognosis, as patients with concurrent autoimmune phenomena might warrant closer follow-up.

## 4 Conclusion

This case report documents the efficacy of an early, intensified combination therapy incorporating TCZ in an adult patient with rapidly progressive deep morphea complicated by multiple aPL seropositivity. The patient’s rapid clinical improvement, coupled with objective MRI evidence of disease regression, confirms the benefit of aggressive, targeted anti-fibrotic intervention during the active inflammatory phase of severe LoS. Notably, this outcome was achieved without waiting for first-line therapy to fail, suggesting that early use of biologics like TCZ may be warranted in select high-risk cases. Additionally, the identification of a rare overlap between localized scleroderma and aPLs in this case provides a valuable example for clinicians: it emphasizes the importance of individualized assessment and therapy, including immunomodulatory and antithrombotic strategies, in managing atypical and severe presentations of morphea.

## Data Availability

The original contributions presented in the study are included in the article/supplementary material. Further inquiries can be directed to the corresponding author.
